# Process evaluation of an environmental health risk audit and action plan intervention to reduce alcohol related violence in licensed premises

**DOI:** 10.1186/s12889-016-3123-9

**Published:** 2016-05-28

**Authors:** Annie Williams, Simon C. Moore, Claire Shovelton, Laurence Moore, Simon Murphy

**Affiliations:** DECIPHer, Cardiff School of Social Sciences, Cardiff University, 1-3 Museum Place, Cardiff, CF10 3BD UK; Violence Research Group, School of Dentistry, College of Biomedical and Life Sciences, Cardiff University, Cardiff, CF14 4XY UK; MRC/CSO Social and Public Health Sciences Unit, University of Glasgow, Lilybank Gardens, Glasgow, G12 8RZ UK

**Keywords:** Alcohol, Violence, Night-time economy, Licensed premises, Environment, Health and safety, Process evaluation

## Abstract

**Background:**

Alcohol-related violence is associated with licensed premise environments and their management. There is a lack of evidence for effective interventions to address these, and there are significant barriers to implementation. This study aims to understand how development and implementation processes can facilitate intervention reach, fidelity and receipt and therefore provides key process data necessary to interpret the results of the randomised controlled trial conducted in parallel.

**Methods:**

A process evaluation, embedded within a randomised controlled trial. Intervention development and implementation were assessed via focus groups (*n* = 2) and semi-structured interviews (*n* = 22) with Environmental Health Practitioners (EHPs). Reach and fidelity were assessed via routinely collected intervention data, which was was collected from 276 licenced premises across Wales, UK. Case study semi-structured interviews with licensed premises proprietors (*n* = 30) explored intervention receipt.

**Results:**

Intervention co-production with senior EHPs facilitated organisational adoption and implementation. Training events for EHPs played an important role in addressing wider organisational concerns regarding partnership working and the contextual integration of the intervention. EHPs delivered the intervention to 98 % of intervention premises; 35 % of premises should have received a follow up enforcement visit, however EHP confidence in dealing with alcohol risk factors meant only 7 % of premises received one. Premises therefore received a similar intervention dose regardless of baseline risk. Intervention receipt appeared to be greatest in premises with an existing commitment to prevention and those in urban environments.

**Conclusions:**

The study suggests that a collaborative approach to the development and diffusion of interventions is associated with high levels of organisational adoption, implementation and reach. However, the lack of enforcement visits represents implementation failure for a key mechanism of action that is likely to influence intervention effectiveness. To be effective, any future intervention may require a longer implementation period to develop EHP confidence in using enforcement approaches in this area and multiagency enforcement support, which includes the police, to deliver an adequate intervention dose.

## Background

The close attention paid by policy makers to the night time economy in the United Kingdom (UK) [1] reflects the rising numbers of city-centre licensed premises [2], the increasing levels of concern about violence in such premises [3], and the emergence of a culture shared with many other countries where excessive alcohol consumption and alcohol-related disorder are tolerated [4]. Alcohol-related harm is now estimated to cost the UK in excess of £21 billion a year [5]. Health costs form a significant part of this [5–7] and alcohol-related harm in night time environments are responsible for an estimated 70 % of all unscheduled Accident and Emergency (A&E) attendances [5–8]. This has led to urgent calls for interventions to reduce levels of alcohol-related harm in the UK and further afield [9, 10].

Alcohol-related violence in the night-time economy is influenced by numerous factors, many of which can be found in and around public houses, bars and clubs across the world [9–11]. These influences include crowding, poor premise upkeep, inappropriate layout, low lighting levels, excessive noise, poor ventilation, irresponsible alcohol promotions, inadequate responsible server training, poor in-house policy and poor adherence to licensing conditions [10–12]. ‘Broken Windows Theory’ (BWT) [13, 14] suggest that addressing such factors can reduce alcohol-related violence, as motivation to offend is informed by situational cues such as graffiti, vacant buildings and broken or boarded windows that indicate an absence of capable guardians and a want of social order defined as “organised responses to crime, delinquency and allied forms of deviant and/or socially problematic behaviour that are actually conceived of as such” [15] and so “identify convenient places to commit crime” [16]. In this way, social control is a central component in understanding the way violence arises in night time environment. Within this, distinctions are made between 'formal' and 'informal' social controls; the former relating to interventions enacted by agencies of the state usually under the auspices of legal authority (e.g. the police), while the latter is concerned with the regulatory and social ordering function performed by citizens (including bar staff and private security).

A systematic review of factors associated with alcohol-related harm concluded that interventions targeting responsible beverage service training, community mobilisation, improved premises policies and enforcing existing licensing laws may be most effective [12]. Despite this, rigorous randomised controlled trials (RCT) of the effectiveness of licensed premises interventions to address environmental factors remain limited.

In light of this and following the Medical Research Council Framework for complex evaluations [17], a feasibility trial of a premise-level intervention to reduce alcohol related violence in licensed premises in Wales was piloted [18]. The intervention consisted of an audit of premise risk-factors and a subsequent action plan to address identified factors. Although study findings demonstrated that the intervention had potential, they also suggested formal, rather than informal, enforcement in this policy-led intervention would be more likely to produce positive effects [19]. It was concluded that any subsequent intervention should be implemented by authorities who hold regulatory powers and who are trained to conduct work-place risk assessments to reduce crime and disorder-related violence in work settings. The Environmental Health Agency in Wales was approached and and agreement was reached that they would collaborate on the implementation of an All Wales Licensed Premises Intervention (AWLPI). The intervention that was delivered was called SMILE (Safety Management in Licensed Establishments’) and a randomized controlled trial methodology was adopted.

This paper presents results from an embedded process evaluation that aimed to explore intervention development, implementation and the subsequent reach, fidelity and receipt of the intervention. In so doing it provides key process data that can facilitate an understanding of main trial outcomes and establishes what works, for whom and in what context.

## Methods

### Research design

A longitudinal process evaluation that examined the development and implementation of the intervention. This was nested within a RCT comparing violence in intervention and control groups of licensed premises (public houses, nightclubs or hotels with a public bar) with a history of violence, ethical approval was provided by the Cardiff University Dental School’s Research Ethics Committee.

### Intervention

**Fig. 1:**
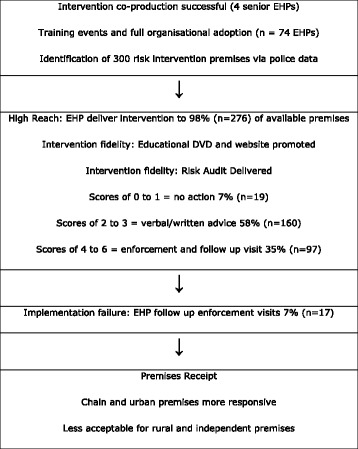
Implementation of SMILE

The SMILE intervention (Fig. 1) was co-produced with senior EHPs and consisted of a risk-audit and action/enforcement plan for premises identified as at risk for violence based on previous incidents in police data. This was delivered by EHPs and supported by a DVD and web-site and provided educational films promoting awareness and due diligence among premises staff. The risk-audit completed by EHPs aimed to identify and highlight areas of premises operation associated with increased levels of alcohol-related violence. The Audit was developed from the literature documenting features of premises that contribute to harm and were shaped so that opportunities to inform change were consistent with the statutory powers EHPs have available to them.

EHPs work mostly with the Health and Safety at Work Act. While licensed premises are covered by the Health and Safety at Work Act there is also specific legislation for the alcohol industry, the 2003 Licensing Act and it is the Licensing Act that is mostly used to regulate licensed premises. For these reasons, EHPs had little direct experience of working with licensed premises and it was therefore necessary to develop intervention materials that worked within EHPs statutory competencies but was also able to translate the available evidence and theory in this area into a workable format.

The Health and Safety at Work Act played a fundamental role in SMILE development as under this legislation all businesses with five or more employees are obliged to have a written policy that describes how risks are identified and managed, all businesses are expected to conduct risk assessments and take reasonable actions to reduce risk. This risk assessment therefore provides the point through which formal control (i.e. Health and Safety at Work Act and the Licensing Act) can operate to increase informal governance whereby premise managers work to identify areas in which harm, including alcohol-related harm (e.g. dealing with intoxicated and disorderly customers), might arise and what can be done to minimise those risks. These risks should be reviewed regularly and employees are expected to be aware of what measures are in place. Dissemination is through formal induction processes for new employees and regular refresher or training sessions for existing staff. The Health and Safety at Work Act therefore provides an important opportunity to manage risk in licensed premises and encourages appropriate informal governance across the entire premises environment.

The risk audit examined 11 operational domains: records management, visibility and lighting, health and safety, surveillance, noise and communication, risk planning, door management, managing people, managing disorderly patrons, staff training, incident reporting and glassware policy. Each of the 11 sections included a Risk Control Indicator (RCI) score. The RCI is a standard instrument used by EHPs to record their perceived level of risk in the area under scrutiny. RCI scales ranged from zero to six: a score of one represents a situation where the EHP believed that no further improvements were possible (based on current legislation and guidance), scores of two and three represent situations where verbal or written feedback may be appropriate, four or higher denotes situations where enforcement action, an improvement or prohibition notice, is required. Zero denotes “not applicable”. For enforcement actions, EHPs were required to perform a follow up visit to check compliance. Each operational domain required EHPs to record any action they had taken (none, verbal advice, written advice, the issuing of an improvement notice or a prohibition notice). EHPs also recorded whether a follow up visit had been completed.

Organisational adoption was promoted by three training days for EHPs held across Wales. The training days consisted of presentations by medical consultants, who highlighted the extent and severity of injuries related to violence in the night time economy; academic staff, who presented the rationale, aims and objectives of the SMILE evaluation and senior EHPs, who presented and discussed the SMILE risk-audit in detail. Seventy-four EHPs attended and SMILE audit tools and contact details for 300 allocated intervention premises were distributed to Local Authority Environmental Health teams after the training event.

### Sampling and recruitment

The process evaluation explored SMILE development and implementation with senior EHPs and a sample of EHPs who delivered SMILE. Four senior EHPs involved in intervention development were invited to take part verbally; information sheets and consent forms were completed at one of the regular research group meetings. Seventy-five EHPs took part in SMILE delivery. Of these, one practitioner from each Local Authority in Wales was randomly sampled and invited to participate in the process evaluation. The sample included three practitioners who had not attended SMILE training (*n* = 22). EHP recruitment took place by email or telephone, with information and consent forms supplied and returned electronically, no refusals were received.

A RCT with embedded economic and process evaluations in 600 premises was undertaken. Premises were randomly assigned (1:1), balanced for history of violence, opening hours and Local Authority EHP capacity, to either the intervention group or usual practice. All premises with a history of violence in the 12 months preceding the project were eligible. EHPs were masked to usual practice premises (but aware of intervention practices). To explore intervention receipt and its relationship to normal practice within premises, a sample of premises were drawn from control and intervention premises. As wide a range of study premises as feasibly possible were included, and although study resources precluded any selection by randomisation, care was taken to ensure premises were selected from across Wales and represented the stratification criteria. Recruitment continued until theoretical saturation was achieved. This resulted in data collected from 30 premises (Table 1).

**Table 1:** Licensed premises sample

Premises	Area (Wales)			Location and Violence Strata					
				Urban		Town/Fringe		Rural	
	North	West	South East	High	Low	High	Low	High	Low
Total (*N* = 30)	4	2	24	8	6	3	10	1	2
Intervention (*N* = 16)	2	1	13	5	3	1	6	0	1
Control (*N* = 14)	2	1	11	3	3	2	4	1	1

### Data collection

Senior EHPs (*n* = 4) took part in two separate focus groups. One focus group, conducted after the intervention development phase, explored the role of the senior EHPS in the co-production of SMILE and its impact on organisational adoption. The second, which took place after SMILE delivery, investigated managerial perspectives on implementation.

Semi-structured telephone interviews were conducted with EHPs (*n* = 22). The interviews explored EHP knowledge of and role in addressing alcohol-related violence in licensed premises before SMILE began, the role of the training day in facilitating organisational adoption of SMILE and the experiences of EHPs during implementation of the intervention. Further detail of intervention reach, fidelity and dose was assessed through routine data collected as part of the intervention audit and action plan.

Interviews with premise managers/owners (*n* = 30) were conducted face to face when possible (*n* = 18) or by telephone (*n* = 12). The interviews collected information about managers/owners experiences of alcohol-related violence and usual action taken to address this issue. In addition intervention premise managers/owners were asked about receipt of SMILE. All semi-structured interviews were held shortly after the intervention phase had ended.

### Analysis

Interviews and focus groups were recorded, transcribed, anonymised and entered into password-protected files before analysis using NVivo 10. The first phase of analysis involved transcript scrutiny and the categorisation of data into dominant themes determined a priori and explored by semi-structured interviews. Audit risk scores and subsequent actions were used to assess the reach, fidelity and dose of the intervention as delivered

## Results

### Intervention development and professional competencies

SMILE was adopted as an Environmental Health project after a series of meetings with senior managers who identified a clear link between the intervention and existing professional skills and competencies. However, reaction of the wider environmental health practitioner body to SMILE highlighted concerns with dealing with alcohol as a new area in which they lacked skills and experience.

Figure 1 provides an overview of the implementation of SMILE.

During early meetings between academics and organisational managers, description of the feasibility study led to recognition that SMILE would map well onto current EHP work practices, ‘*I thought “that sounds like some the work that we do, so how come we are not joined up and doing something together?”* (Senior EHP2). These meetings also improved academic understanding of the organisational context and the remits of routine EHP practice *‘investigating accidents.... umm, they educate, they work in partnership with groups, other stakeholders who have an interest in public health or the health of the environment’* (Senior EHP1).

Subsequent discussion revolved around the design and delivery of SMILE. Organisational managers suggested that Health and Safety EHPs should implement the intervention, as these specialists were likely to have gained useful experience in RIDDOR (Reporting of Injuries, Diseases and Dangerous Occurrences Regulations, 2013) investigations, and in *‘an [earlier] alcohol and violence project, not necessarily for Licensed Premises, but in book makers, uhh night clubs....restaurants as well* (Senior EHP1). Further deliberation about possible changes needed to adapt the risk-audit intervention to the environmental health context resulted in the agreement of senior EHPs to co-produce SMILE to ensure the audit mapped onto existing working practices as closely as possible.

For the wider EHP community, although all Local Authorities had chosen to participate in SMILE, half of the EHPs (*n* = 11) interviewed felt inclusion had been imposed. Despite such perceptions of a ‘forced adoption’, a recognised barrier to implementation [19, 20], most EHPs anticipated that SMILE may progress their work into a new legitimate arena *‘we are a responsible authority for licensed premises I don’t think that we are finding out enough about what is happening at these premises… and if they are not managing violence at their premises then I think that is something that we should be doing’* (EHP19).

The greatest practitioner concern at this stage was environmental health involvement in SMILE as the sole delivery agency ‘*licensing might have been better placed to deal with this’* (EHP22). Some local authority EHPs addressed this by consulting licensing colleagues and making joint visits to premises. Additional concerns were associated with confidence in dealing with alcohol as a new area *‘we would deal with violence but not specifically alcohol-related violence and certainly we wouldn’t have had a role with regards alcohol-related violence between customers’* (EHP8).

Contrary to managerial belief, levels of previous experience in addressing work-related violence amongst EHPs were found to be low. A quarter of practitioners maintained they had little experience in this type of work*: ‘it wasn’t something that we really, we didn’t look at really’* (EHP1). Of those who had experience, eleven had gained little within licensed *premises ‘I wouldn’t say they was a massive emphasis on it, you know we may look at, if we were going to a licensed premises’* (EHP17). Of the remaining five practitioners, only one had worked on a local project similar to AWLPI `*through local knowledge and police statistics, I targeted out problem areas and visited all of those’* (EHP5). Others had worked with or as part of local authority multi-agency teams, with involvement ranging from occasional *‘they meet on a regular basis, now as environmental health officers we don’t sit on that on a regular basis’* (EHP12) to fuller integration *‘we apply this scheme in [X] … it basically looks at all the issues in terms of NTE, sort of drinking related, alcohol related problems, under age sales, all these kinds of things’* (EHP10). It was also discovered that EHPs had conducted little ARV work through RIDDOR *‘to be honest I don’t recall ever having one’* (EHP13). Despite these findings, there was no suggestion that EHPs felt lack of experience in these fields would pose barriers to project adoption and participation and these issues were subsequently addressed during training for organisational adoption.

### Organisational adoption

Following intervention co-production, EHPs from every Welsh Local Authority attended training days that aimed to familiarise them with the intervention and ensure they held the requisite knowledge and skills to deliver the intervention.

Seventy-four EHPs attended the training days, such high attendance promoted the organisational reach of SMILE. The majority of EHPs felt the research team gave *‘good insight to the background, you know to see where it comes from, the thought behind it and, you know, what you hoped to achieve from doing the project’* (EHP2), whilst clinicians offered ‘*the visual sort of displays of injuries that were, in A&E from the, from the medical practitioner that was on site, was, was quite… Well it was hard hitting’* (EHP5). Overall the training appeared to raise motivation to deliver the intervention ‘*and it did, you know, sort of get me sort of more enthusiastic about trying to tackle it’* (EHP5). The co-production of SMILE also proved important in facilitating adoption and implementation *‘because our colleagues had been involved in it from the start that did help because you know you weren’t sort of actually preaching to people who had no sort of feedback from peers’* (EHP10), and producing an intervention that met existing skill sets ‘*the actual forms I think are pretty self-explanatory in terms of you know filling them in, obviously you know we are used to going on site and taking various different forms with us you know’* (EHP17). Even for EHPs who did not attend the training *‘I had a look through [the risk-audit] myself and any questions that I had about it I could ask my colleagues and they would sort of clear it’* (EHP6).

Despite the generally positive response, some concerns remained about the licensing agency exclusion *‘the violence and the procedures around that with regards to CCTV and all that would have been better suited to licensing’* (EHP18) and ‘*it would have been nice to have had the feedback and the input from the licensing officer side of it’* (EHP15)*,* with a minority concerned as ‘*we don’t want to step on their [licensing] toes!’* (EHP18)*.* This highlighted the importance of agency boundaries in maintaining work practices and relationships [21, 22], and supported the need for EHPs to adapt the intervention to further assimilate it into their routine practice [23].

Most EHPs were also impressed by the supportive web-site and films during training days *‘I felt that I could say to the publicans oh you know it does give you a lot of good information, you know such as Challenge 25. And so I thought there was a lot of good information on the DVD,’* (EHP2). However two voiced concerns about one section of one film *‘as a team we were slightly horrified by the contents of the training video which you know indicated that premises should put staff into harm’s way in order to protect customers, which is just abhorrent, it is just everything that we would advise against’* (EHP8): these practitioners did not promote the videos extensively.

### Intervention implementation

The co-production approach appeared to produce an acceptable and usable intervention with audit data and reports of SMILE delivery from both EHPs and premise managers/owners suggesting significant intervention reach and high levels of fidelity for the risk audit. However, concerns regarding the fidelity of action plan implementation and subsequent intervention dose, due to low levels of enforcement visits emerged. Low levels of concerns about the relevance of the intervention to all premises were also found.

### Intervention reach

Although the delivery of SMILE by EHPs led to significant intervention reach (98 %), a number of premises closed down before the intervention was delivered, and a small number proved uncontactable or refused to participate. Of the 300 original intervention premises selected, 92 were ineligible owing to premises closure and four refused. Within refusals, one was attributed to an ongoing prosecution, another landlord declined because he had recently been part of a similar project, a further because he felt that SMILE selection reflected poorly on the premises: ‘*he got quite upset about it and I think [research team member] got involved as well and you know he had to send you know an apology out’* (EHP21). The final refusal was based on disbelief: *‘Why are you here? You know I can’t remember the last time there was even an incident’* (EHP22). This feeling was shared by managers of many premises in rural or quiet locations.

Overall, EHPs delivered the intervention to 69 % of the initial sample. As per protocol, replacement premises for premises closures and refusals were allocated. In total EHPs delivered SMILE to all but four of the available intervention premises that were open and could be contacted. Such a high implementation rate is consistent with studies in other areas involving statutory partners and strengthens the evidence that agents without legal powers are inferior when compared with those that do have legal power [24, 25]. Indeed, nearly all managers consulted reported feeling obligated to co-operate with authorities: *‘it’s going to happen, so may as well go with it’* (DPS 342).

### Implementation fidelity

EHPs delivered the risk audit as prescribed. Most EHPs found the risk-audit easy to use ‘*the layout and everything was all sort of easy to use and I thought that it was fine*’ (EHP1). All referred to the audit guidance during scoring, and some used it exclusively ‘*we just utilised your guidance*’ (EHP9), but many drew on previous experience and/or interaction with premises staff ‘*how responsive they are to what you are talking about. There is no point in serving notices on somebody who is very willing and very happy to put things in place*’ (EHP7). Individual approaches to risk audit delivery varied: some practitioners used more open attitudes ‘*they were always looking at what you were writing and stuff, and it was quite easy for then for me to explain to them with the tick boxes*’ (EHP11) than others ‘*I would fill in the pro-forma and then come back and do my scores back in the office, so I wouldn’t say to them at the end because obviously that would be quite confrontational*’ (EHP7). Few problems in promoting the website and DVD were reported although the two practitioners who had held concerns about the advice in one film had distributed DVDs with warnings to ignore the section.

**Fig. 2:**
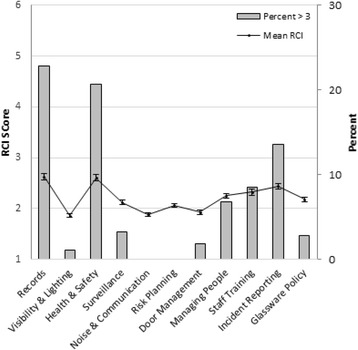
Risk Audit scores

Risk audit data results and resulting action/enforcement plans provide a measure of the dose delivered to each premises. Risk control indicator scales across 11 domains ranged from zero to six, where a score of zero denotes "not applicable" (these scores were dropped from summary statistics), scores of two to three would suggest verbal or written advice and scores of four to six would warrant enforcement actions and follow up visits. Figure 2 highlights results from the Risk Control Indicator Scores and shows that the areas requiring most attention included record keeping (including written risk assessments), health and safety, and incident reporting.

**Fig. 3:**
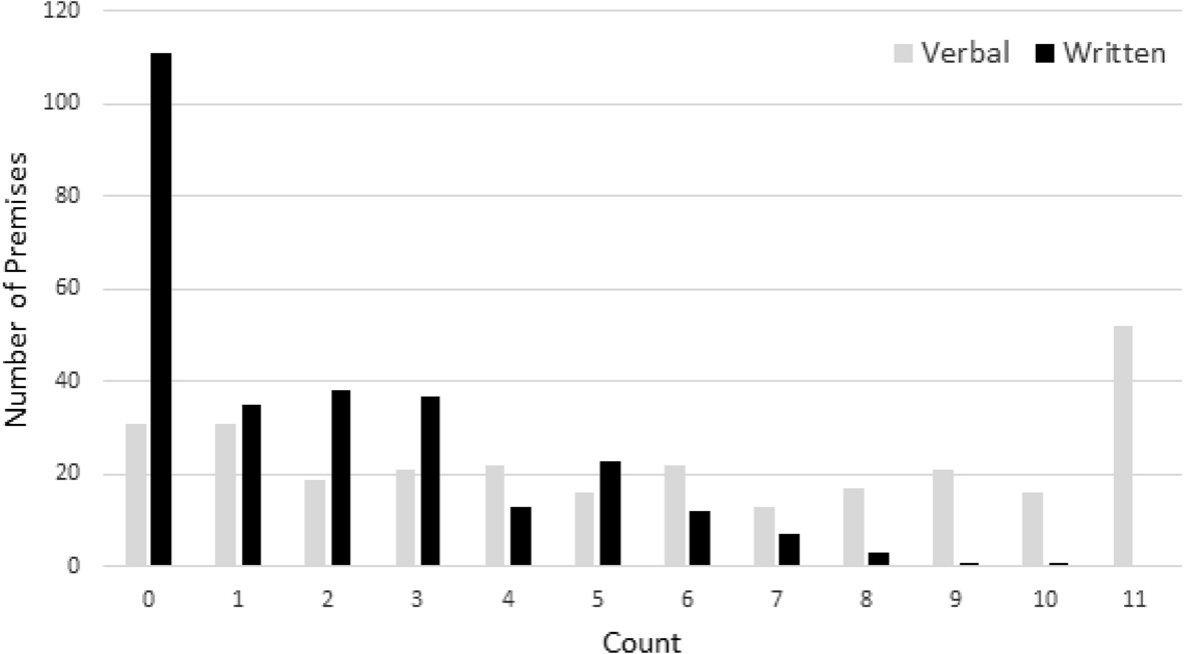
Audit action plan count of advice given by type

Figure 3 shows the count of advice given by type of advice (verbal or written). Each premises could be given verbal or written advice for each of the eleven areas covered by the audit. In total, premises could receive a maximum of eleven items of verbal and eleven items of written advice. This histogram presents the number of premises by the total number of verbal and written items received. These data suggest that verbal advice was used more often and for more areas of interest in the audit than written advice.

The majority (57.6 %) received verbal and supporting written advice. Significantly, although 35 % should have received an enforcement action and follow up based on their risk audit scores, only 18 (7 %) of premises received one, with EHPs only reverting to more formal procedures when actions were not made or legislation had been compromised*’with issues then which I did pick up on it was written advice, I didn’t need to take any further enforcement action after the written advice and re-visiting them, they had done all the, you know they had done all the issues which I had picked up*’ (EHP6). Most follow-up visits were related to health and safety checks (*n* = 13) and staff training (*n* = 13).

### Intervention receipt

Half of the EHPs felt premises reacted positively to SMILE ‘*all of them were accommodating and you know quite a few of them were actually happy then …because …maybe they hadn’t had an inspection or visit off us for you know a number of years*’ (EHP6). Some practitioners ascribed this to the statutory nature of visits, others to pre-existing positive relationships with premises. Owners supported this view, ‘*if you are doing it with the local authority you know that you have covered everything and there is nothing that you have missed out yourself’* (LP336). In general, EHPs reported that managers of large, often chain premises, tended to react more positively as SMILE fitted in well with established routines ‘*some of the bigger ones … where they have got good procedures in place and well trained managers and a well-run place that are receptive to it‘*(EHP3) with poorer responses often coming from long-time owners of smaller pubs ‘*they said ‘have you ever worked in a pub’ and I said ‘no’, ‘well’ they said ‘well there we are, I have worked in a pub for 30 years, I know exactly what goes on here, I know exactly what to look for”* (EHP3).

Such descriptions of mixed SMILE reception were confirmed by the accounts of 16 intervention premise owners/managers (n =7 chain, *n* = 9 independent). For their part, most were convinced issues were already being addressed adequately by established in-house policies, practices and training *‘all I have got to do is phone head office, and they are like there with an answer’* (LP347). However, it is worth noting that this opinion was not universal: four managers of chain premises, including two where SMILE had identified no area(s) of concern, felt that the intervention had been beneficial as it has raised personal awareness of alcohol-related violence and refocused attention on the issue ‘*it pushes me to the right direction, that you have got to be focused on these types of things you know’* (LP84).

Furthermore, some proprietors noted that SMILE had encouraged maintenance of present standards and updated knowledge *‘fresh eyes, do you know what I am saying, so I mean and anything new or anything I mean it is like if I have been here six years and … maybe, she will come and say oh why don’t you try it that way because it is a fresh idea you know. Sometimes so anything new really is always a good idea I think’* (LP124). For independents, despite one premise reacting very positively, ‘*I don’t have the information and there are lots of different things she brought with her, booklets and that as well but especially that DVD….., it has just highlighted different zones. ….and I say ok I am taking time out here now and I am doing this and showing the staff this as well, and it is all good isn’t it really. It is positive’* (LP131), a significant number were unhappy at the idea of receiving SMILE. A couple of owners insisted the intervention had no place in their premises and represented an unwelcome burden *‘I suppose if you hadn’t ticked a lot of the boxes then yes it would be an eye opener and it would be of value. But personally speaking I had a lot of the work already done’* (LP179). Others participated without complaint, gave SMILE little thought afterwards, and did not see increased EHP involvement positively *‘once is bad enough!’* (LP100).

Participants managing urban premises also appeared as more responsive to the intervention, with all agreeing that alcohol related violence was an established problem, and most feeling the situation had worsened in recent years with escalating drugs use compounding matters ‘*It has got a lot worse over the last few years because there is so much, there is so much drugs in the valleys now it’s sort of an accumulation of the two’* (LP174). Despite this, a minority working in town environments described alcohol related violence as something that occurred outside of their premises or responsibility *‘people walking down from* [X] *Street two of them seen each other…. history of a feud between them and they started fighting outside. And it has ended up our doormen have got involved because it is literally our doorstep and other people have got, and it just escalated and escalated and it was my doorman that got into trouble for it - for stepping off their door’* (LP336) or as a consequence of individuals drinking elsewhere; either at other premises or through preloading. The inference that premises prefer to distance themselves from customer violence, was reinforced by comments such as *‘The biggest issue is once people have become intoxicated is having the foresight, as managers and door staff, to remove those people from the venue’* (LP28). Such comment supports evidence that violence and aggression are often displaced onto the street [23] and strengthens calls for some premises to be given stronger support to help them take responsibility for their role in alcohol related violence.

### Intervention reconfiguration

Most EHPs felt SMILE had been worthwhile, had had a beneficial impact on the knowledge and practices of EHPs, and fitted with routine work practices. Some suggestions to improve targeting, enforcement and sustainability were also offered.

There was wide agreement amongst EHPs that SMILE fitted the organisational context well ‘*seems to be certainly something that would tie in naturally with the Health and Safety at Work Act’* (EHP20) with the importance of sustaining and integrating this work into professional practice rather than being a ‘one off’ intervention stressed *‘I know from like, from personal experience and they have got good intentions for that, the next few months and then it sort of slips off and it goes off their agenda and then something else’* (EHP13).

In contrast, owners and managers were surprised to find EHPs delivering SMILE. Regardless of this, after receiving SMILE most felt EHPs had a role in assessing risk factors given their knowledge and statutory powers *‘you would make double sure that, you know on that night, you know, it is one of those things that you shouldn’t work like this, you do you just say I will go and double check,*’ (LP 308). EHPs agreed that statutory powers encouraged actions within premises *‘these interventions highlight the issue to the managing agent and as a result, as is generally the case when we book an assessment, the first thing those in control of the business does is review their own risk assessments on that subject’* (EHP8).

Further reflection produced some suggestions for SMILE reconfiguration. A major concern stemmed from the data used to identify participant premises. Although virtually all EHPs felt the premises visited had been representative of their areas, over half felt the wrong premises had been selected ‘*some of the ones perhaps that I would have classed as being a problem pubs weren’t within that list….. I know that obviously it is a random controlled trial …but the ones that we know we have had issues with, it would be nice to know, well if we do something with that pub will it make that much of a difference’* (EHP5). Many respondents agreed and felt SMILE effectiveness would have been increased if delivery had been confined to premises whose managers had little experience or knowledge of alcohol-related violence and how to minimise it: *‘the ones that weren’t part of a chain, independent, no controls, ones with no, no real understanding’* (EHP22), or which were known to have larger levels of problematic behaviours and violence. Many EHPs suspected that the police violence records were unreliable ‘*you said that a lot of the data came through from the police but when I checked up, certainly on the one pub they had nothing, there was nothing against it whatsoever’* (EHP7). Police violence records were also criticised for associating incidents with nearby premises ‘*three of the pubs in particular, they have called the police for problems outside the premises, you know it is nothing to do with their premises but yet they are, you know they are brought up on the list’* (EHP3) and for not differentiating between police attendance to prevent trouble as oppose to managing on-going violence *‘people believed they were serving under age but they were very pro-active in wanting to kind of clean that up…and I think that is why they had a higher number of police incidents’* (EHP18).

Post-implementation, questions about the legitimacy of environmental health being the sole agents of SMILE delivery remained, and the feeling that SMILE should have included licensing officers had strengthened ‘*a lot of things that sort of licensees have to do which ties in with this and [licensing] have got a lot of hands on knowledge of individual premises, individual licensees’* (EHP4). This view was shared by a couple of proprietors ‘*if a policeman had come in and done that study with me I would have found it more appropriate than the EHP woman coming in to do it’* (LP22) who were concerned that SMILE drew more agencies into a field already negatively affected by increased funding for police and local authorities in licensing over the last decade *‘I think that perhaps bringing more bodies into the kind of …..maybe….Yeah I think that, as I say at the moment there is way too much conditions on licenses and stuff like that’* (LP157). SMILE also introduced or reinforced appreciation of the value of multi-agency work, especially for EHPs with similar earlier experience *‘people like the health board, the police, you know fire service, other agencies … you know getting everybody interested’* (EHP17). EHPs also maintained *‘it is important to forge these links with everybody … so that they can actually, you know raise concerns and perhaps you know change license conditions and deal with all these things rather than you know do it in isolation’* (EHP10)*.*

## Discussion

Given the lack of rigorous international evidence identifying effective statutory interventions for preventing alcohol-related violence, these findings provide important process data for the interpretation of the forthcoming paper on the effectiveness of SMILE. Such randomised controlled trials of unproven interventions are important from an ethical point of view in conditions of social equipoise [24] to identify potential benefits or harms. Without process data, it will not be possible to explain why the intervention may or may not have been effective, for whom and in what circumstances [15, 25]. In itself this study adds to our knowledge of how to implement interventions addressing factors in and around licensed premises and highlights key learning that has generalizable implications for the future development and implementation of interventions drawing on broken windows [13, 14] and social control theory [15, 16] delivered by statutory bodies across cultures and contexts.

It is clear that the statutory nature of the intervention, drawing on formal social control mechanisms, facilitated high levels of reach, particularly when compared to results from the previous feasibility trial and reflects similar international studies of statutory social control interventions [26, 27]. However this finding needs to be considered alongside knowledge of the high numbers of closures that occurred before the intervention could be delivered, instances of premise inaccessibility, and the very small number of premises that refused to take part. Such findings demand some consideration of heavier enforcement policies for externally driven formal social control, especially for the minority of refusers for whom a multi-agency approach involving wider authorities may encourage full participation. In this, it reflects international findings that highlight the importance of the police in delivering interventions drawing on social control approaches and broken windows theory [28]. Independent premises seemed to have the strongest need for such an intervention. The risk-audit identified multiple areas of concern in three quarters of the independent intervention premises included in the process evaluation, with SMILE acceptability lower and receipt poorer in such premises, whose managers tended to see the intervention as an added burden that they lacked the resources to respond to. Such cases demand consideration of more complex formal social control interventions that include additional resources/support to promote formal and informal social control within premises. There were also concerns of whether the police data were robust enough to identify and reach the most at-risk premises. Such anxieties led to associated questions about whether the intervention was targeted at those in most need of it.

In terms of intervention fidelity and the subsequent dose, some EHPs demonstrated lack of confidence in dealing with alcohol related violence at the start of the project, and although the training appeared to allay concerns, many practitioners subsequently relied on advice and guidance rather than using enforcement to deliver the intervention regardless of the risk scores they allocated. In this, the audit was used as a motivational rather than enforcement tool and the action plans were not delivered with fidelity. At the end of the project many EHP concerns about dealing with alcohol-related violence remained. This may well change as responsibility for alcohol related violence is further embedded in organisational practice but, significantly, there was strong support for a multiagency approach, including licensing officers and police, to support enforcement and full intervention implementation. This highlights the need for the development and evaluation of multi-agency rather than single agency interventions in this area as recommended by the World Health Organisation [29] and, in terms of research design, it suggests the need for a longer period of adoption for new practices to be embedded before any evaluation is undertaken.

Such implications should be considered in light of a number of study limitations. As highlighted above the first of these concerns bias. The loss to follow up of premises through closures and a small but significant number of premises who were either inaccessible or refused to take part in the study may be associated with unmeasured response bias. Additionally, although there was an attempt to sample a diverse range of premises types for the process evaluation, it maybe that participation was associated with an additional level of response bias. In terms of design, it should be noted that premises level data was collected from a relatively small number of bars and clubs compared to the number receiving the intervention due to pragmatic and cost considerations. Although this allowed an examination of the processes of receipt and implementation, process data for a greater number or indeed all premises would provide a more comprehensive understanding. In a similar way, observational work examining audit delivery and premises receipt would have provided additional understanding. Finally, although the pragmatic nature of the study did not allow it, a longer period of pre-intervention research, where existing practices and assessment and review of new practices may have facilitated understanding and an opportunity to address implementation barriers.

## Conclusion

Alcohol is bought and consumed in premises in most cities around the world, reflecting the ubiquity of a global alcohol industry and highlighting the need for interventions internationally to address alcohol related violence. It is clear that the statutory nature of the intervention led to high reach. Such approaches appear a necessary pre-requisite for licenced premises interventions. Study findings suggest that environmental health is an agency that possesses the potential infrastructure, expertise, statutory powers and skills to deliver such interventions in the UK, but it was also clear that a multi-agency approach and longer term integration into professional practice is necessary. In the absence of this and the failure of a key mechanism of action there are concerns whether the intervention was of sufficient intensity to promote effectiveness.

## Abbreviations

A&E, accident and emergency; AWLPI, all wales licensed premises intervention; BWT, broken windows theory; EHP, environmental health officer; RCI, risk control indicator; RCT, randomised controlled trial; SMILE, safety management in licensed environments
